# Synthesis and Characterisation of ASA-PEEK Composites for Fused Filament Fabrication

**DOI:** 10.3390/polym14030496

**Published:** 2022-01-26

**Authors:** Belén Palacios-Ibáñez, José J. Relinque, Daniel Moreno-Sánchez, Alberto S. de León, Francisco J. Delgado, Ramón Escobar-Galindo, Sergio I. Molina

**Affiliations:** 1 Departamento de Ciencia de los Materiales e Ingeniería Metalúrgica y Química Inorgánica, IMEYMAT, Facultad de Ciencias, Campus Río San Pedro s/n, Universidad de Cádiz, 11510 Puerto Real (Cádiz), Spain; danielmoreno.sanchez@uca.es (D.M.-S.); alberto.sanzdeleon@uca.es (A.S.d.L.); fjavier.delgado@uca.es (F.J.D.); sergio.molina@uca.es (S.I.M.); 2 Departamento de Física Aplicada, Escuela Politécnica Superior, Universidad de Sevilla, C/Virgen de África 7, 41011 Sevilla, Spain; rescobar1@us.es

**Keywords:** thermoplastic polymers, composite materials, fused filament fabrication, mechanical properties, thermal properties

## Abstract

In this paper, a series of polymer composites made from acrylonitrile-styrene-acrylate (ASA) and poly (ether ether ketone) (PEEK) were manufactured. ASA acts as a polymer matrix while PEEK is loaded in the form of micro-particles that act as a reinforcing filler. The composites were compounded by single screw extrusion and then, different specimens were manufactured either via injection moulding (IM) or fused filament fabrication (FFF). Two different types of PEEK (commercial and reused) in different concentrations (3 and 6 wt.%) were tested and their influence in the mechanical, structural, and thermal properties were studied. It was observed that reused PEEK enhanced the stiffness and tensile strength and thermal stability of the composites both, for injected and printed specimens. This evidences the suitability of these composites as potential candidates as novel materials with enhanced properties following an approach of circular economy.

## 1. Introduction

Circular economy has emerged in the last years as an alternative which allows to change the linear take-make-waste process [1,2]. While the conventional fabrication model implies transforming raw materials into final products which will be disposed at the end of their lifecycle, the circular economy approach considers that the generated waste can be reused as raw material in the same or another fabrication process. The products at the end of their lifecycle may be exploited instead of definitely losing their value, optimising the resources and making the manufacturing processes more sustainable.

Additive manufacturing (AM) processes involve a number of techniques where objects are manufactured upon a 3D computer aided designed model by the deposition of a material in a layer-by-layer approach [3]. The AM techniques are aligned with the circular economy approach, since they generate less waste than conventional subtractive techniques [4,5]. Moreover, they can easily fabricate parts with complex geometries and high added value in short-runs [6], saving energy and reducing operative costs [7,8,9].

AM technologies can be classified in seven different categories, depending on their physicochemical working principle [10]. Among these categories, material extrusion (ME) consists in the deposition of a molten material (typically thermoplastics or composites) through a heated nozzle at a temperature above its melting point. ME techniques include filament fused fabrication (FFF), which is the most widespread AM technology, since it is inexpensive and its fabrication conditions are milder compared to other AM technologies involving the manufacturing of metals or ceramics [11]. FFF allow the use of recycled plastics or other natural products as wood or cork, enabling more sustainable processes [11,12,13,14,15].

The most used materials for FFF are high molecular weight polylactic acid (PLA) and acrylonitrile-styrene-butadiene (ABS) terpolymer. These materials in their molten state present a proper viscoelastic behaviour (typically at 200–250 °C) that allows their processing via FFF [16,17]. Nevertheless, 3D-printed objects with PLA are relatively brittle and cannot be used above 60 °C [18,19]. ABS is preferred for most engineering applications, even though it has poor weather resistance due to the olefinic bonds present in the butadiene core, which is susceptible to UV degradation. This causes a decrease in the mechanical properties and yellowing of the material [20]. Acrylonitrile-styrene-acrylate (ASA) has emerged as an alternative that overcome these issues. The substitution of the butadiene rubber by an acrylate elastomer improves the weathering shown by ABS [21]. The mechanical performance of ASA can be further improved by combining it with other polymers or fibres [22].

On the other hand, poly (ether ether ketone) (PEEK), is a technical thermoplastic with high mechanical and thermal performance (Young’s modulus 4000 MPa, tensile strength 100 MPa, glass transition temperature 153 °C, melting temperature 343 °C). This polymer is widely used in engineering applications that require a high mechanical performance that cannot be typically achieved with ASA or ABS. Moreover, PEEK also exhibits high chemical resistance [23,24]. Its major drawback lies in the higher processing temperatures required, compared to most of the thermoplastics and its high cost.

In this work, ASA-based composites containing PEEK suitable for FFF were prepared. PEEK was used as an additive, in the form of particles in a compromise to obtain materials with enhanced mechanical properties at an affordable cost. Due its high melting point, PEEK can be considered as a ceramic filler, so it is considered that a composite is obtained, instead of a blend of polymers. Two different PEEK grades were used as fillers: a commercial grade PEEK powder (P) and a reused PEEK powder (CP) generated from machining waste, following a circular economy approach. The composites were prepared by single screw extrusion and subsequently processed by injection moulding (IM) and FFF. Their mechanical properties were determined and the influence of the printing orientation and raster angles, as key parameters in the eventual tensile properties of the printed parts, were assessed as well. These results were correlated with structural characterisation by scanning electron microscopy (SEM) of the fracture surface of tested tensile specimens. Finally, the thermal behaviour of the composites was studied by termogravimetry analysis (TGA) and differential scanning calorimetry (DSC), pointing out the influence of PEEK in the thermal stability of the composites.

## 2. Materials and Methods

### 2.1. Materials

ASA (*ASA LI 941 NC* supplied by *LG,* Seoul, South Korea, and distributed by *Biesterfeld Ibérica S.L.*, Barcelona, Spain) was used as the polymer matrix. Virgin PEEK (P) particles (D_p_ < 25 µm) were supplied by *Victrex*, Lancashire, UK, and distributed by *Policomplex S. L.*, Valencia, Spain (*PEEK 150 XF)*. CoPEEK powder (CP) collected from machining waste with a wide granulometry ranging from micrometres to millimetres was supplied by *Bieglo GmbH*, Hamburg, Germany. The CP particles were sieved using a vibratory sieve shaker (*AS200*, supplied by *Retsch GmbH*, Haan, Germany) and only the fraction 45–125 µm was used as a filler. All the materials were oven dried for at least 3 h at 80 °C and used without further modifications to prepare the composites.

### 2.2. Synthesis of Composites

The synthesis of the composites was carried out by melt extrusion using a benchtop single screw extruder (*Noztek Pro Filament* Extruder supplied by *Noztek*, Shoreham-by-Sea, UK). ASA, CP, and P were introduced at different concentrations in the hopper, as presented in Table 1. The materials were processed at 260 °C, at a rotation speed of 60 rpm, and a filament of 1.75 ± 0.5 mm diameter was obtained. To improve the dispersion of PEEK in the ASA matrix, the filaments obtained were air cooled, oven dried again for at least 3 h at 80 °C, chopped to pellets (3–5 mm in length), and reprocessed in a second extrusion cycle under the same conditions. Part of the filaments obtained were chopped again to obtain pellets suitable for injection moulding (IM).

### 2.3. Preparation of Samples

**Figure 1:**
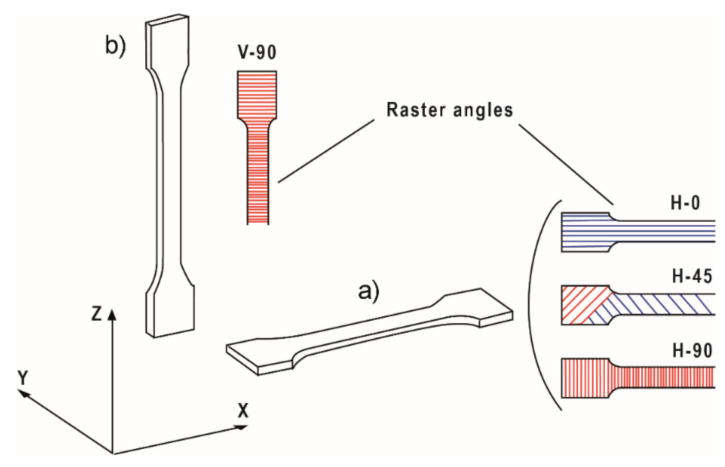
Orientations and raster angles of the different tensile test specimens. (**a**) Horizontal orientation, with raster angles parallel to the building axis, X, (0°, H-0), ±45° (H-45), and perpendicular (90°, H-90); (**b**) vertical orientation with the raster angle perpendicular to the building axis, Z, (V-90).

Tensile testing specimens were manufactured by IM and FFF according to UNE-EN ISO 527 standard (type 1BA specimens). Pellets of the different composites were injected using a *Babyplast 6/10P* (supplied by *Cronoplast S.L.*, Abrera, Spain). The heat profile was 230/240/240 °C (plastification chamber/injector/die) and pellets were two times compressed at 70 and 50 bar, respectively, upon injection in the mould. On the other hand, 3D-printed tensile testing specimens were printed using a *Raise 3D Pro2* printer (supplied by *Raise 3D*, Irvine, CA, USA). The .gcode file for the test specimens manufacturing was generated using the freeware *IdeaMaker.* Samples were manufactured modifying the orientation and raster angle of the infill to determine their influence in the mechanical properties of the materials with different building directions. Figure 1 summarises graphically the different orientations and raster angles tested. The printing speed was fixed to 25 mm/s to ensure the proper printing of all the specimens, in particular V-90, which are rather slender. The rest of the printing conditions were left by default for ASA materials, according to *IdeaMaker*: 0.2 mm layer height, 100% infill, printing temperature 235 °C and 100 °C bed temperature.

ASA and 3 wt.% PEEK composites were printed in horizontal and vertical orientation and the raster of the infill was varied from 0° to 90°. The raster angle is that between the axial position of the specimen test and the infill. In the case of ±45° infill, the specimens are built alternating sequential layers at 45° and −45° (see Figure 1).

### 2.4. Characterisation Methods

Structural and compositional characterisation of the composites was performed by SEM and Fourier-transformed infrared spectroscopy (FTIR). The fracture surface of the tensile testing specimens was observed by SEM. SEM images were taken using an *Auriga CrossBeam* microscope (supplied by *Zeiss*, Oberkochen, Germany) and a *Scios 2 DualBeam* microscope (supplied by *ThermoFisher Scientific*, Waltham, MA, USA). The microscopes were operated in SEM mode (secondary electrons images) under low voltage (3–15 kV). The samples were sputter-coated with Au using a high resolution sputter coater *208 HR* (supplied by *Cressington*, Watford, UK), prior to observation in SEM. FTIR analyses were carried out in a *Bruker Alpha* spectrometer (supplied by *Bruker*, Billerica, MA, USA) in the attenuated total reflectance (ATR) mode with a spectral resolution of 4 cm^−1^. Spectra were acquired from FFF printed monolayers of ASA, ASA-3P and ASA-3CP, and from P and CP powder.

The mechanical properties were evaluated by tensile testing of normalised specimens of the composites. For that purpose, a universal tester *AGS-X* (supplied by *Shimadzu*, Kyoto, Japan) was used. The specimens were all tested at 1 mm/min and the results were the average of at least five experiments, in agreement with UNE-EN ISO 527.

Thermal characterisation of the composites was performed by TGA and DSC. Thermograms were obtained using a *Q600 SDT* analyser (supplied by *TA Instruments-Waters LLC*, New Castle, DE, USA) by heating the samples from 25 °C to 900 °C at 10 °C/min under inert N_2_ flow (100 mL/min).

## 3. Results and Discussion

### 3.1. Mechanical Properties of the Composites

**Figure 2:**
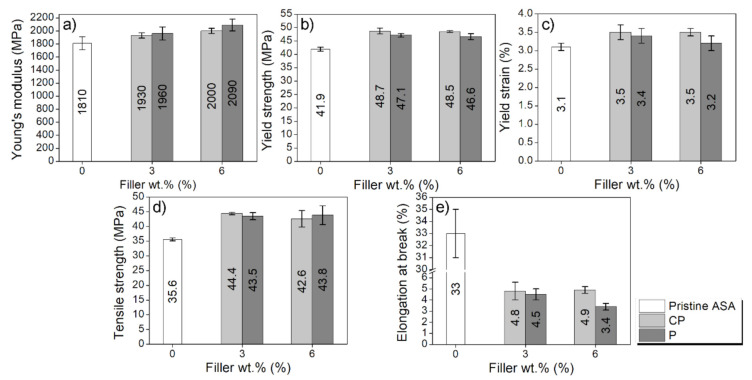
Mechanical properties of the injected pristine ASA (white bars), ASA-CP (light grey bars), and ASA-P (dark grey bars) with confidence intervals. (**a**) Young’s modulus; (**b**) yield strength; (**c**) yield strain; (**d**) Tensile strength, and (**e**) elongation at break.

First, normalised specimens for tensile testing were manufactured by IM and tested in order to find out a trend in the mechanical behaviour of the composites. The results for the injected specimens are presented in the graphs depicted in Figure 2. The corresponding stress–strain curves have been included in Figure S1 as Supplementary Material.

According to the results, the stiffness (evaluated as Young’s modulus, Figure 2a) and the strength (evaluated as yield strength, Figure 2b, and tensile strength, Figure 2d) improved for ASA-PEEK composites, compared to pure ASA. In particular, the Young’s modulus increases up to 15% while the yield strength and the tensile strength increase 16% and 25%, respectively. Previous reports show that ASA can improve the mechanical properties of polymer matrices such as SAN [20], PMMA [25], PVC [26], or PC [27] by blending, using high amounts of ASA. However, in this work, ASA is used as a matrix while PEEK (either CP or P) is acting as a filler (like a ceramic particle, since PEEK does not melt under the conditions used in this study). Hence, the composites consists of the dispersion of PEEK particles in an ASA matrix, rather than a blend of polymers. ASA-CP and ASA-P composites behave quite similar and exhibit comparable properties and trends when increasing the PEEK amount, even though they have different particle size. This supports that CP can be used instead of P, evidencing that a PEEK as a waste can be used as feedstock instead of purchasing an expensive material as it is P.

ASA-CP and ASA-P composites do not exhibit any improvement in mechanical properties when increasing the PEEK concentration. The presence of fillers in polymers implies a reduction in the free volume and, hence, limits the polymeric chain mobility. In principle, the mechanical properties are expected to increase when the filler content is higher [28]. However, this general trend is also affected by the dispersion and the compatibility (i.e., interface adhesion) of fillers with the polymeric matrix. In this case, in the absence of a proper blend between ASA and PEEK, an increase of PEEK content does not likely enhance the interface adhesion with ASA, together with an increase of agglomerations of PEEK [29]. As a result, the behaviour presented by the 3 and 6 wt.% PEEK composites is not significantly different.

Since an enhancement in the mechanical properties from 3 to 6 wt.% was not observed, only composites containing 3 wt.% PEEK (either P or CP) were printed via FFF. This permits to reduce the amount of PEEK used, while it is also expected to avoid clogging problems in the nozzle while printing [30].

**Figure 3:**
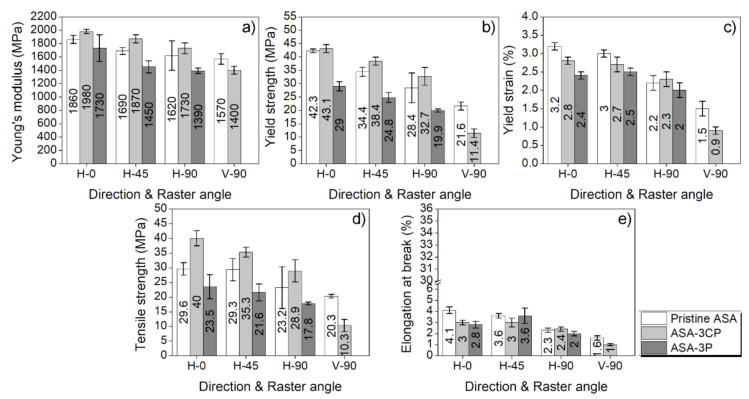
Mechanical properties of the FFF printed ASA (white bars), ASA-3CP (light grey bars), and ASA-3P (dark grey bars) with confidence intervals. (**a**) Young’s modulus; (**b**) yield strength; (**c**) yield strain; (**d**) Tensile strength, and (**e**) elongation at break. The composites were printed in horizontal and vertical orientation, using raster angles of 0, 45 and 90° for the first orientation (H-0, H-45, and H-90, respectively), and 90° for the second one (V-90).

Both fillers were tested to check out the behaviour of the composites when processed by FFF. The mechanical properties of ASA-3CP and ASA-3P composites printed by FFF in several orientations and raster angles (see Figure 1) are shown in Figure 3. The corresponding stress–strain curves have been included in Figure S2 as Supplementary Material.

The composites manufactured by FFF exhibit different properties when the raster angle is varied [31]. As observed in Figure 3, the best mechanical performance is expected in parts printed for H-0 specimens. In this case, the tensile stress is held by each printed road rather than the bonds between roads which would support a minimum load. When the raster angle is varied, these bonds are forced to withstand part of the tensile stress as well as the roads. Therefore, for H-0 samples, the fracture is dominated by the material failure whilst for H-45 and H-90 samples, delamination events happen through the bonds between roads, which eventually leads to material failure [32]. Similarly, the printing orientation plays a key role in the mechanical performance of FFF printed parts. Since the only possible raster angle in the vertical orientation is 90° (V-90 samples), the roads are pulled perpendicularly during the tensile test, thus the main stress is exerted on the inter-layer bonds [33,34].

**Figure 4:**
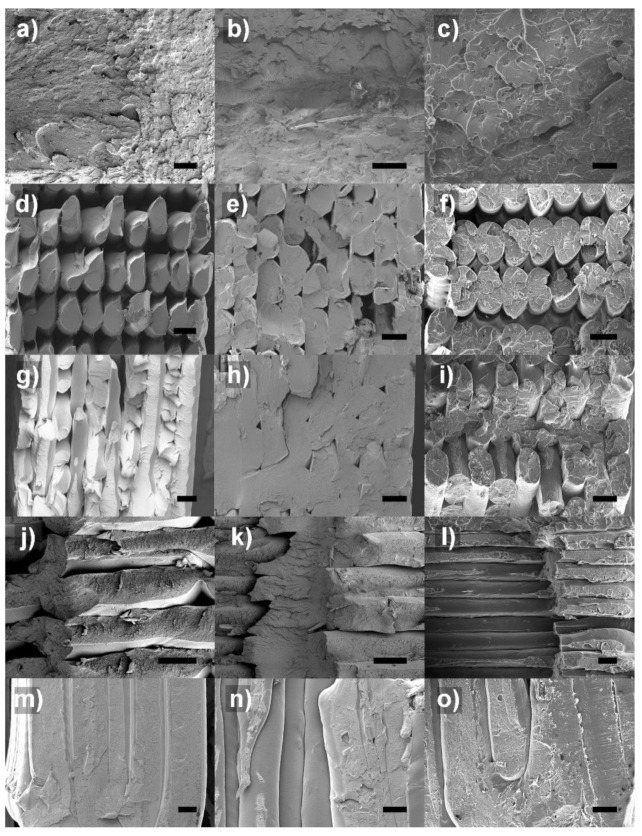
SEM images of the fracture surface of composites manufactured by (**a**–**c**) IM; (**d**–**f**) FFF-H0; (**g**–**i**) FFF, H45; (**j**–**l**) FFF, H-90, and (**m**–**o**) FFF, V-90. ASA ASA-3CP, and ASA-3P are presented in the left, middle, and right column, respectively. The scales bar stands for 200 µm in all cases.

In this work, the composites are tested at different orientations and raster angles to identify the actual performance of 3D-printed objects under different building directions. The results presented in Figure 3 are coherent with the theoretical trend discussed and previous works found in the literature [35]. In general, the mechanical properties decrease when the raster angle is increased (i.e., the mechanical properties of H-0 are higher than those of H-90) and the vertically printed parts exhibit the weakest mechanical performance, as expected. The stiffness (Figure 3a) and strength (Figure 3d) of ASA-CP increase up to 11% and 35%, respectively, when compared to pristine ASA, while the elongation at break values decrease minimally (Figure 4e). These results are in agreement with previous reports using ABS or PLA-based composites [36]. However, vertically printed (V-90) ASA-PEEK composites are weaker than pristine ASA. In particular, V-90 ASA-P composites were extremely brittle. This did not allow to perform adequately the testing of its mechanical properties, since the test specimens broke when tightening them within the clamping jaws of the tester. Hence, these results are not represented in Figure 3. V-90 ASA-CP specimens were properly tested, even though they are more brittle than pristine ASA. This fact may be related to the different morphology of CP and P, as it will be discussed below.

### 3.2. Structural and Compositional Characterisation of the Composites

The fracture surface of the composites was analyzed by SEM to obtain more information about the structural behavior of these materials, as depicted in Figure 4. Figure 4a shows the fracture surface of the pristine ASA manufactured by IM. It presents the characteristic aspect of a ductile fracture, exhibiting a rough surface caused by the plastic deformation of ASA before failure. On the contrary, ASA-3CP (Figure 4b) and ASA-3P (Figure 4c) composites prepared by IM present smoother surfaces. This can be correlated to the brittle behaviour previously observed for the composites. In the case of the FFF printed composites, all the raster angles can be easily noted. Since all of the specimens underwent a brittle fracture, the surfaces are more even than those observed by IM. SEM results evidence that, as stated before, the higher the raster angle, the more likely delamination can happen between roads of material [37]. The separation of roads is barely noticeable in H-0 samples but it gradually increases for H-45 and H-90 specimens. In the case of H-90, the fracture follows an axial path (instead of a straight crack, perpendicular to the tensile axis) and abrupt jumps can be observed along the fracture surface.

ASA-3CP FFF printed composites, present some differences in the surface between the roads of the fracture surface (Figure 4e,h,k,n). These features, which can also be observed in the sample prepared by IM (Figure 4b), are not observed in pristine ASA, and present a characteristic roughness, so they are likely the CP particles. CP possess a higher particle size than P, so it may have formed a binding structure between the roads and layers of ASA when printing, which would explain the improvement in mechanical properties observed either in FFF samples. This is neither observed for ASA-3P composites (Figure 4c,f,i,l,o), since the P particles have a significantly smaller average particle size. The fracture surface of ASA-3P exhibits a faceted texture in the shape of an irregular honeycomb structure. This is particularly clear for the IM sample (Figure 4c), while it gradually less perceptible in H-0, H-45, and H-90 samples prepared by FFF (Figure 4f,i,l,o). This feature observed in the ASA-3P samples suggests that the filler could have acted as a nucleating agent for fractures, and the observed facets are formed after failure. This may be the reason behind the poor mechanical performance of these composites. On the contrary, in ASA-3CP composites, the CP particles seem to participate in the fracture mechanism as an actual reinforcing filler.

SEM images from the filaments were included in Figure S3 as Supplementary Material. Whilst some voids were observed around CP and P particles, since the diameter of the FFF extruder is lower than that of the injection nozzle, the formation of those imperfections within the deposited material during FFF printing is minimal when roads are deposited, as observed in Figure 4. This would explain the absence of porosity within the printed parts, beyond the gaps between printed roads.

**Figure 5:**
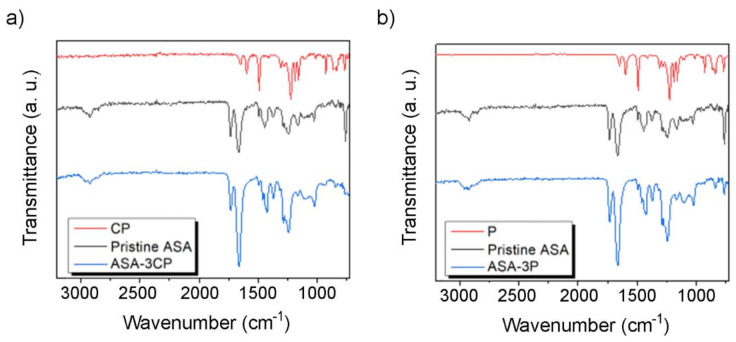
ATR spectra of (**a**) CP, pristine ASA, and ASA-3CP, and (**b**) P, pristine ASA, and ASA-3P.

Chemical analyses of the composites were carried out by ATR and the results are presented in Figure 5. Composites containing CP and P exhibit practically coincident spectra, indicating that the quality of the CP obtained as a by-product is similar than the one of pure PEEK powder (P). Then, the spectra of the composites exhibit signals corresponding both to ASA and PEEK. In these two cases, the spectra acquired also are rather similar. This indicates that the PEEK particles are homogeneously distributed within the ASA-3P and ASA-3CP composites. This effect can also be observed in the band at 1240 cm^−1^, corresponding to the C−O stretching, that are shifted towards lower values, which could be caused by supramolecular interactions between ASA and PEEK.

### 3.3. Thermal Characterisation of the Composites

**Figure 6:**
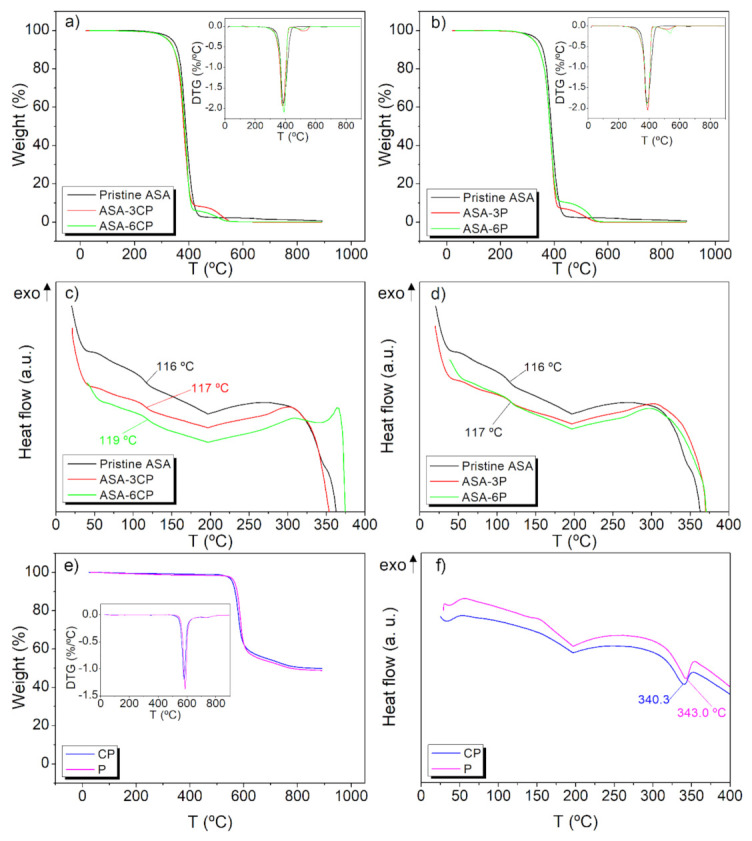
TGA thermograms of pristine ASA and (**a**) ASA-CP composites, and (**b**) ASA-P composites, (the corresponding DTG curves are included, respectively, in the insets); DSC curves of pristine ASA and (**c**) ASA-CP composites, and (**d**) ASA-P composites; (**e**) TGA thermograms of CP and P (with the corresponding DTG curves in the inset), and (**f**) DSC curves of CP and P.

TGA and DSC analyses were carried out on pristine ASA, CP, P, and the composites containing 3 and 6 wt.% of either CP or P. The results are presented in Figure 6 together with the first derivative curves of TGA, i.e., derivative thermogravimetry (DTG). For pristine ASA, there is one single degradation step, with a maximum rate at 386 °C, according to the DTG curve (Figure 6a). ASA fully degrades at 450 °C. These results are in agreement with previous works found in the literature [38]. The thermograms corresponding to either CP and P show two decomposition stages (Figure 6e). According to the literature, the first one, ranging between 580–590 °C is caused by random chain scissions of ether and ketones, producing phenol and other aromatic compounds (benzene or dibenzofuran) as by-products. However, carbonyl bonds are more stable and able to resist these temperatures. The degradation of these bonds is produced in a second degradation peak at ca. 730 °C. Above this temperature, a carbonaceous char, ranging from 46 to 50% of the original mass, is obtained as a residue [39].

Regarding the DSC analysis, the literature describes for pristine ASA two glass transitions. The first one (not shown) at ca. −45 °C, corresponding to the rubber part of the terpolymer and the second one corresponding to the poly (acrylonitrile-styrene) block at a temperature higher than 100 °C [18,31,33]. As expected, the obtained curve for the pristine ASA observed in Figure 6 exhibits the second glass transition at 116 °C. No other thermal transitions are observed, due to the amorphous character of the terpolymer. DSC curves of either CP or P show their melting peak ranging 340 °C. Other authors report the presence of a glass transition and an exothermic crystallisation, due to its semi-crystalline behaviour, before observing the endothermic melting [40,41]. In this work these events are not perceptible, probably because these composites have undergone several heating and cooling cycles during the manufacturing, which may have induced a high crystallinity degree [42]. The ASA-PEEK composites present a similar degradation profile than pristine ASA, but including the second characteristic transition of PEEK. All the composites were fully degraded at the end of the experiment, i.e., the material was fully degraded at 400–500 °C (Figure 6a,b). 

No clear trends were observed when increasing the filling rate and no influence of the filler in the thermal stability was observed. The DSC profiles of the composites and ASA were practically coincident, except for a slight increase in the glass transitions and a subtle increase in the temperature of decomposition (Figure 6c,d), so it can be concluded that the presence of CP or P slightly improves the thermal properties of ASA.

## 4. Conclusions

In this work, new ASA-PEEK suitable for IM and FFF was prepared. The composites retained the good processability of pristine ASA as well as its thermal stability, with improved stiffness and strength, even when low amounts of PEEK (i.e., 3 wt.%) were used. This allows to have a new family of composite materials at an affordable cost, since PEEK is an expensive polymer material. Moreover, the use of CP shows the possibility of further reducing the cost of these materials, in the framework of circular economy. ASA-CP composite exhibited better properties than ASA-P, since a reinforcing structure was formed within the composite, likely due to the higher particle size, as evidenced by tensile testing and SEM analyses. CP exhibited a similar chemical behaviour than P, indicating that it can replace virgin PEEK in these applications, which enables the development of new PEEK composite materials valid for FFF with a low environmental impact, expanding the current FFF portfolio of materials. The surface modification of the PEEK particles with compatibilising agents, as well as the use of PEEK fibres instead of particles are contemplated as future work, to achieve better mechanical properties.

## Figures and Tables

**Table 1:** Prepared composites and synthesis conditions.

Composite	Filler	Concentration (wt.%)	Test Specimen Preparation
Pristine ASA	-	-	IM, FFF
ASA-3CP	CP	3	IM, FFF
ASA-6CP		6	IM
ASA-3P	P	3	IM, FFF
ASA-6P		6	IM

## Data Availability

Not applicable.

## Supplementary Materials

The following supporting information can be downloaded at: https://www.mdpi.com/article/10.3390/polym14030496/s1, Figure S1: Stress-strain curves of pristine ASA, ASA-CP, and ASA-P composites prepared by IM; Figure S2: Stress-strain curves of pristine ASA, ASA-3CP, and ASA-3P composites prepared by FFF in the following orientations and raster angles (a) H-0; (b) H-45; (c) H-90, and (d) V-90; Figure S3: SEM images of filaments used to FFF print (a) pristine ASA; (b) ASA-3CP, and (c) ASA-3P, showing some porosity within the filaments of ASA-3CP and ASA-3P.
