# Supplementary material for: Synthesis and Characterisation of ASA-PEEK Composites for Fused Filament Fabrication
Source: Polymers (Basel). 2022 Jan 26;14(3):496. doi: 10.3390/polym14030496 (PMC8838854; doi:10.3390/polym14030496)
Supplement: Supplementary file 1 [file polymers-14-00496-s001.zip › polymers-1528850-supplementary.pdf]

polymers

MDPI

*Supplementary Material*

# Synthesis and Properties Assessment of ASA-PEEK Composites Suitable for Fused Filament Fabrication

# Synthesis and Characterisation of ASA-PEEK Composites for Fused Filament Fabrication

**Belén Palacios-Ibáñez [1,*], José J. Relinque [1,*], Daniel Moreno-Sánchez [1], Alberto S. de León [1], Francisco J. Delgado [1], Ramón Escobar-Galindo [2] and Sergio I. Molina [1]**

[1] Departamento de Ciencia de los Materiales e I. M. y Q. I., IMEYMAT, Facultad de Ciencias, Campus Río San Pedro s/n, Universidad de Cádiz, 11510 Puerto Real, Spain; danielmoreno.sanchez@uca.es (D.M.-S.); alberto.sanzdeleon@uca.es (A.S.d.L.); fjavier.delgado@uca.es (F.J.D.); sergio.molina@uca.es (S.I.M.)

[2] Departamento de Física Aplicada, Escuela Politécnica Superior, Universidad de Sevilla, C/Virgen de África 7, 41011 Sevilla, Spain; rescobar1@us.es

* Correspondence: belen.palacios@uca.es (B.P.-I.); josejavier.relinque@uca.es (J.J.R.); Tel.: +34-956-01-2028 (B.P.-I. & J.J.R.)

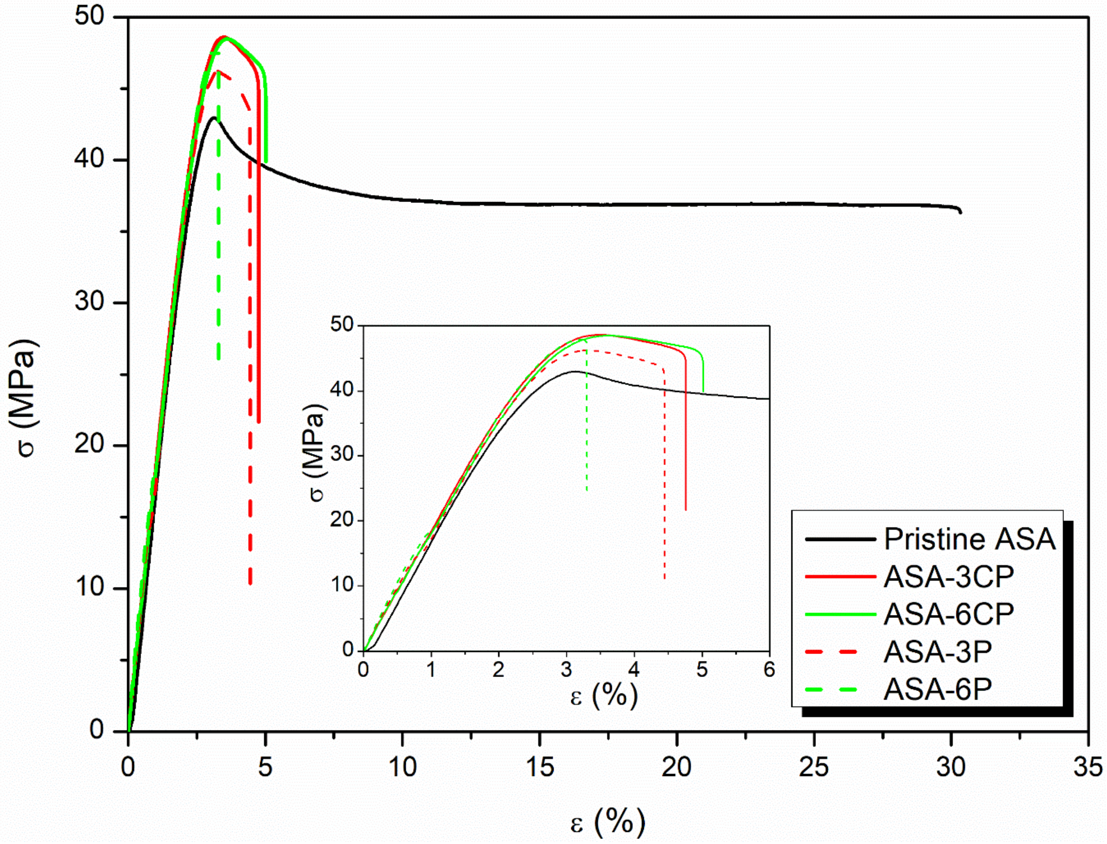


**Figure S1.** Stress-strain curves of pristine ASA, ASA-CP, and ASA-P composites prepared by IM

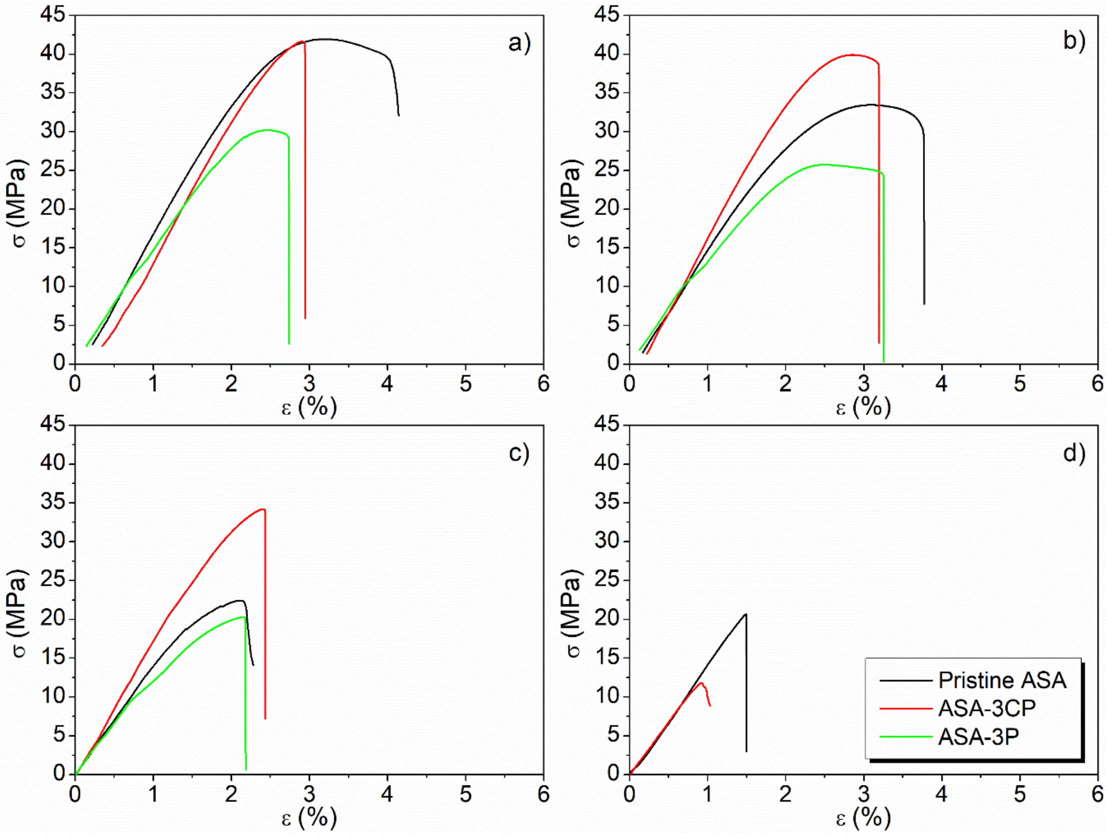


**Figure S2.** Stress-strain curves of pristine ASA, ASA-3CP, and ASA-3P composites prepared by FFF in the following orientations and raster angles **(a)** H-0; **(b)** H-45; **(c)** H-90, and **(d)** V-90.

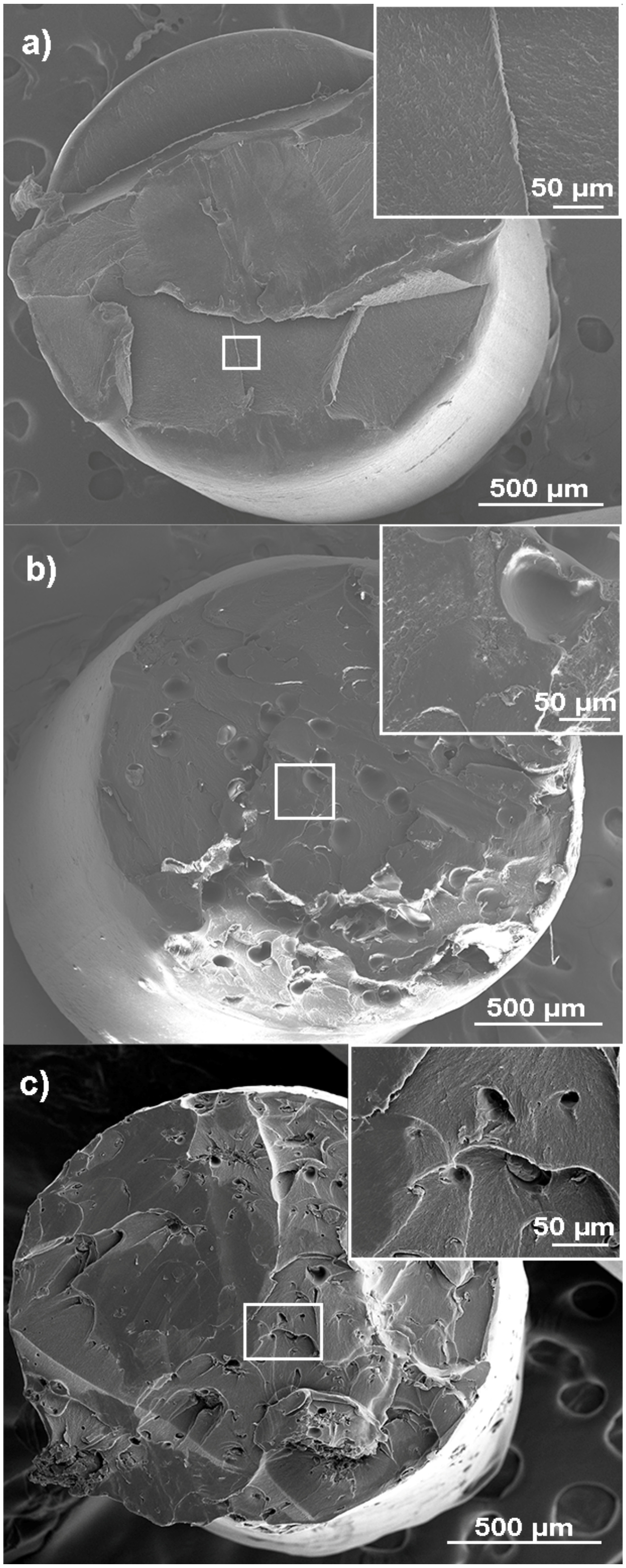


**Figure S3.** SEM images of filaments used to FFF print **(a)** pristine ASA; **(b)** ASA-3CP, and **(c)** ASA-3P, showing some porosity within the filaments of ASA-3CP and ASA-3P.
